# H_2_S catalysed by CBS regulates testosterone synthesis through affecting the sulfhydrylation of PDE

**DOI:** 10.1111/jcmm.16428

**Published:** 2021-03-13

**Authors:** Jing Wang, Jing Wang, Tao Shen, Renyun Hong, Shanshan Tang, Xia Zhao

**Affiliations:** ^1^ Department of Reproductive Medicine Zhongda Hospital School of Medicine Southeast University Nanjing China

**Keywords:** cAMP/PKA, cystathionine‐β‐synthase, hydrogen sulphide, phosphodiesterase, testosterone

## Abstract

Testosterone deficiency resulted in increased mortality in men. Our previous work found that hydrogen sulphide (H_2_S) significantly alleviated the spermatogenesis disorder. To investigate whether H_2_S could regulate testosterone synthesis and the relative signalling pathways. Disorder model of testosterone synthesis was constructed in vitro and in vivo. The cell viability was detected using CCK‐8 method. The concentration of H_2_S and testosterone were examined using ELISA kits. The relative mRNA and protein expression of CBS, PDE4A, PDE8A and proteins related to testosterone synthesis were detected by RT‐qPCR and western blotting. PAS staining was used to detect the inflammatory status of testis. The sulfhydryl level of PDE4A and PDE8A was determined by Biotin Switch Technique. CBS overexpression inhibited while knockdown promoted LPS + H_2_O_2_ induced injury in testosterone synthesis of MLTC‐1 cells, though regulating the level of H_2_S. The LPS + H_2_O_2_ induced inhibition on cAMP and p‐PKA was recovered by CBS overexpression, while addition of the specific inhibitor of PKA had opposite effects. CBS overexpression alleviated the inflammation status in testis and promoted the expression of StAR, P450scc, P450c17 and 3β‐HSD. CBS could also exhibit its protective role through promoting sulfhydrylation of PDE4A and PDE8A. H_2_S catalysed by CBS could recover testosterone synthesis in vitro and in vivo through inhibiting PDE expression via sulfhydryl modification and activating cAMP/PKA pathway.

## 1 INTRODUCTION

Testosterone is the most important reproductive hormone in men, which is mainly synthesized by male testis and plays an important role in the growth, development, reproduction and maintenance of organ function. Testosterone deficiency can seriously affect male reproductive and sexual functions and cause abnormal glucose metabolism and dysfunction of cardiovascular system, resulting in decreased quality of life and increased mortality.^1^, ^2^, ^3^ Studies have shown that the incidence of hypogonadism and low testosterone levels is about 2.1%‐5.7% in men aged 40‐79 years.^4^ For a long time, androgen replacement therapy has been the main means to solve the insufficient synthesis and secretion of testosterone, which can alleviate the dysfunction caused by testosterone deficiency. However, androgen replacement therapy has strict contraindications and needs to be monitored for a long time in the course of treatment, while long‐term use of androgen brings the patients with possible cancer and cardiovascular risks, as well as other adverse reactions.^5^, ^6^ Therefore, it is of great significance to investigate new mechanisms involved in the regulation of testosterone synthesis and find potential new targets for endogenous testosterone regulation.

Hydrogen sulphide (H_2_S), an important gas signalling molecule involved in the regulation of physiological functions, has become one of the research hotspots in recent years. H_2_S in human body mainly catalyses l‐cysteine production by Cystathionine γ‐lyase (CSE) and Cystathionine‐β‐synthase (CBS) and plays a key role in a variety of signal pathways that regulated physiological functions.^7^ In the male reproductive system, CBS is mainly found in the Leydig cells of the testis, and CSE is mainly distributed in Sertoli cells and immature spermatocytes including spermatogonia.^8^ Our previous work found that H_2_S could significantly alleviate the spermatogenesis disorder caused by inflammation and oxidative stress, among which H_2_S derived from CBS might be one of the key factors to maintain testicular function.^9^ H_2_S can play a physiological role by regulating protein post‐translational sulfhydration. H_2_S targets the sulfhydryl group (Cys‐SH) on the side chain of cysteine residues to achieve sulfhydryl (‐SH)‐disulphide bond (‐S‐S‐) conversion, which plays a key regulatory role in a variety of physiological and pathological processes.^10^, ^11^

Phosphodiesterase (PDE) is one of the key targets in regulating the cAMP/PKA pathway of testosterone synthesis. Inhibiting the activity of PDE is of great significance in solving testosterone secretion disorders. Several studies have shown that H_2_S was a non‐specific inhibitor of PDE and participated in the regulation of cAMP‐dependent signalling pathways.^7^ Since PDE proteins contain a large number of cysteine residues, it is speculated that the mechanism by which H_2_S inhibits PDE activity may be related to sulfhydryl sulphide modification.^7^ However, the significance of H_2_S for male reproduction still remains unclear.

In this study, we intended to clarify the new mechanism of H_2_S regulating testosterone synthesis through the modification of PDE sulfhydryl sulphide using mouse Leydig Tumour Cells (MLTC‐1) and inflammation‐induced testosterone synthesis disorder mouse model, aiming at providing theoretical basis for the treatment of testosterone deficiency.

## 2 METHODS

### 2.1 Cell cultures and treatment

MLTC‐1 cells purchased from Cell bank of Chinese Academy of Sciences (shanghai, China) were employed and cultured in Dulbecco's Modified Eagle Medium containing 10% Foetal Bovine Serum, based on the standard protocols. When the cells grew to 80% monolayer, model + H_2_S group was pretreated with H_2_S donor GYY4137 (50 μmol/L, ab142145 from Abcam, CA, USA) for 4 hours while control group and model group were treated with PBS solution. Subsequently, all the groups except control group were added with LPS (10 μg/mL) and H_2_O_2_ (250 μmol/L) for 12 hours to construct disorder model of testosterone synthesis.

### 2.2 Recombinant vector constructs

Mouse CBS cDNA, PDE4A cDNA and PDE8A cDNA clone were designed via GenBank database and synthesized by Nanjing Jiancheng company (Nanjing, Jiangsu, China) and then was sequenced for verification. The CBS‐shRNA (F: 5′‐CCAGUGAGUUCUUCAATT‐3′, and R: 5′‐UUGAAGAACUCACACGTT‐3′) was designed and purchased from Invitrogen company. The construction and identification of recombinant plasmids for overexpression or knockdown were achieved by Nanjing Jiancheng company (Nanjing, Jiangsu, China), packaged into adenovirus (Ad) system and amplified in HEK293 cells for stable transfection in vitro and in vivo. Titers of Ad‐CBS and Ad‐PDE4A, Ad‐PDE8A used in the in vitro studies were determined by qPCR as 1.1 × 10^11^ DRP (DNase resistant particles)/mL, 2.7 × 10^11^ DRP/mL and 8.1 × 10^10^ DRP/mL, respectively.

### 2.3 Animal model constructions

All experimental protocols were approved by the Animal Care and Use Committee of Zhongda Hospital, School of Medicine, Southeast University and performed as per the guidelines for the ethical treatment of experimental animals. The male mice of 8‐week‐old to 10‐week‐old were housed at constant temperature (23 ± 1°C) with 12‐hour light/dark cycle. They were fed according to standard protocol and drank free to water. The in vivo disorder model of testosterone synthesis was constructed by injection of lipopolysaccharide (LPS) (10 mg/kg), 1 hour after which H_2_S donor GYY4137 (50 mg/kg, ab142145 from Abcam, CA, USA) was injected subsequently. The both‐side testes samples were collected 24 hours after injection.

As for overexpressing or inhibiting target gene in vivo, mice were anaesthetized with 2% sodium pentobarbital (40 mg/kg) by routine intraperitoneal injection, and the abdomen was opened and the testicles were removed out. The right‐side testicle was injected with recombinant Ad‐CBS, Ad‐PDE4A, Ad‐PDE8A or Ad‐OE‐NC to overexpresses CBS, PDE4A or PDE8A in the mice (15 μL of Ad‐CBS, 1.1 × 10^10^ pfu per mouse; 15 μL of Ad‐PDE4A, 1.4 × 10^10^ pfu per mouse; 15 μL of Ad‐PDE8A, 0.9 × 10^10^ pfu per mouse). While the left side one was injected with adenovirus vector with plasmid barely containing EGFP at the same dose. The mice were fasted for 7 days and then treated with LPS and GYY4137 as per the above procedure.

### 2.4 Cell viability assay

Cell viability was measured using a CCK‐8 kit (KeyGen Biotech, Nanjing, China). The cells were plated into 24‐well plates at a density of 1 × 10^5^ cell per well and incubated for 24 hours, followed by addition of CCK‐8 solution. The viable cells were quantified at 450 nm by a Microplate Reader (Potenov, Beijing, China).

### 2.5 ELISA assay

The level of H_2_S for mice, testosterone and the activity of PDE was determined via rat‐specific enzyme‐linked immunosorbent assay (ELISA) commercial kits from My BioSource (San Diego, CA, USA).

### 2.6 Western blotting

Western blotting was performed for the detection of protein levels based on as per the reported method with little modification.^12^ The tissue samples or cells were digested by RIPA solution (Solarbio, Beijing, China) for total protein extraction. The collected proteins were separated by SDS‐PAGE and then were transferred a PVDF membrane. Then the membrane was incubated with antibodies against mice StAR (ab133657, 1:1000), P450scc (ab175408, 1:1000), P450c17 (ab125022, 1:1000), 3β‐HSD (ab75710, 1:1000), CBS (ab140600, 1:1000), PDE4A (ab200383, 1:1000), PDE8A (ab109597, 1:1000), PKA (ab75991, 1:1000), p‐PKA (ab75991, 1:1000) and Anti‐β‐actin (ab8226, 1:500) that selected as a control, followed by secondary antibody Goat Anti‐Mouse IgG H&L (HRP) (ab6728, 1:2000). All the antibodies used were purchased from Abcam Co., Ltd. (Cambridge, USA). The density of protein bands was examined using an Image analyser quantitative system.

### 2.7 Biotin Switch Technique (BST)

The collected cell samples were lysed with HEN buffer (250 mmol/L Hepes‐NaOH, pH 7.7, 1 mmol/L EDTA, 0.1 mmol/L neocuproine, 100 µmol/L deferoxamine) and protease inhibitors. The proteins in cell lysates were detected using a BCA kit and were incubated with Biotin‐HPDP (10 mmol/L, Invitrogen, CA, USA) for 3 hours, followed by 20 μL of streptavidin‐agarose beads (Invitrogen, CA, USA) for 18 hours on a roller system at 4°C. The beads were washed twice with ice‐cold HEN buffer solution. The level of sulfhydryl groups in PDE protein was analysed by Western blotting mentioned above and probed with mouse antibody against PDE4A (ab200383, 1:1000) and PDE8A (ab109597, 1:1000).

### 2.8 Real‐time reverse transcription quantitative PCR (RT‐qPCR)

The RNAiso Plus Kit (Takara, Dalian, China) was used to extract the total RNA in tissues, and Reverse Transcription kit (Thermo Scientific, Waltham, USA) was employed to transcribe the RNA into cDNA, after which cDNA product was stored at −20°C. The measurement of mRNA expression level was performed on a real‐time fluorescent quantitative PCR system (Roche Light Cycler96, Roche, Indiana, USA). The RT‐qPCR amplification of cDNA samples was performed under the following conditions: pre‐denaturation at 95°C for 3 minutes (1 cycle); amplification at 95°C for 5 seconds, 60°C for 30 seconds and 72°C for 45 seconds (40 cycles); the last cycle was processed at 72°C for 10 minutes. The expression of the target genes was normalized to the level of internal NAPDH using the 2^−ΔΔCt^ method.

### 2.9 Periodic acid‐Schiff staining (PAS)

The testicles samples were cut into 3 μm slices and then fixed in 10% buffered neutral formalin for histopathological staining. Tissue samples were dehydrated and embedded in paraffin. The slices were mounted on glass layers and stained with Hematoxylin and Eosin (HE).^13^

### 2.10 Statistical analysis

All the data were performed in the form of mean ± standard deviation with each experiment repeated at least three times. Student's *t*‐test was used for comparison between the two groups of data, and Tukey's test for one‐way ANOVA was used for comparison between multiple groups using Prism software version 8.0. The *P*‐value less than .05 was regarded as significantly different.

## 3 RESULTS

### 3.1 H_2_S pretreatment reduced LPS + H_2_O_2_ induced injury in testosterone synthesis of MLTC‐1 cells

**FIGURE 1:**
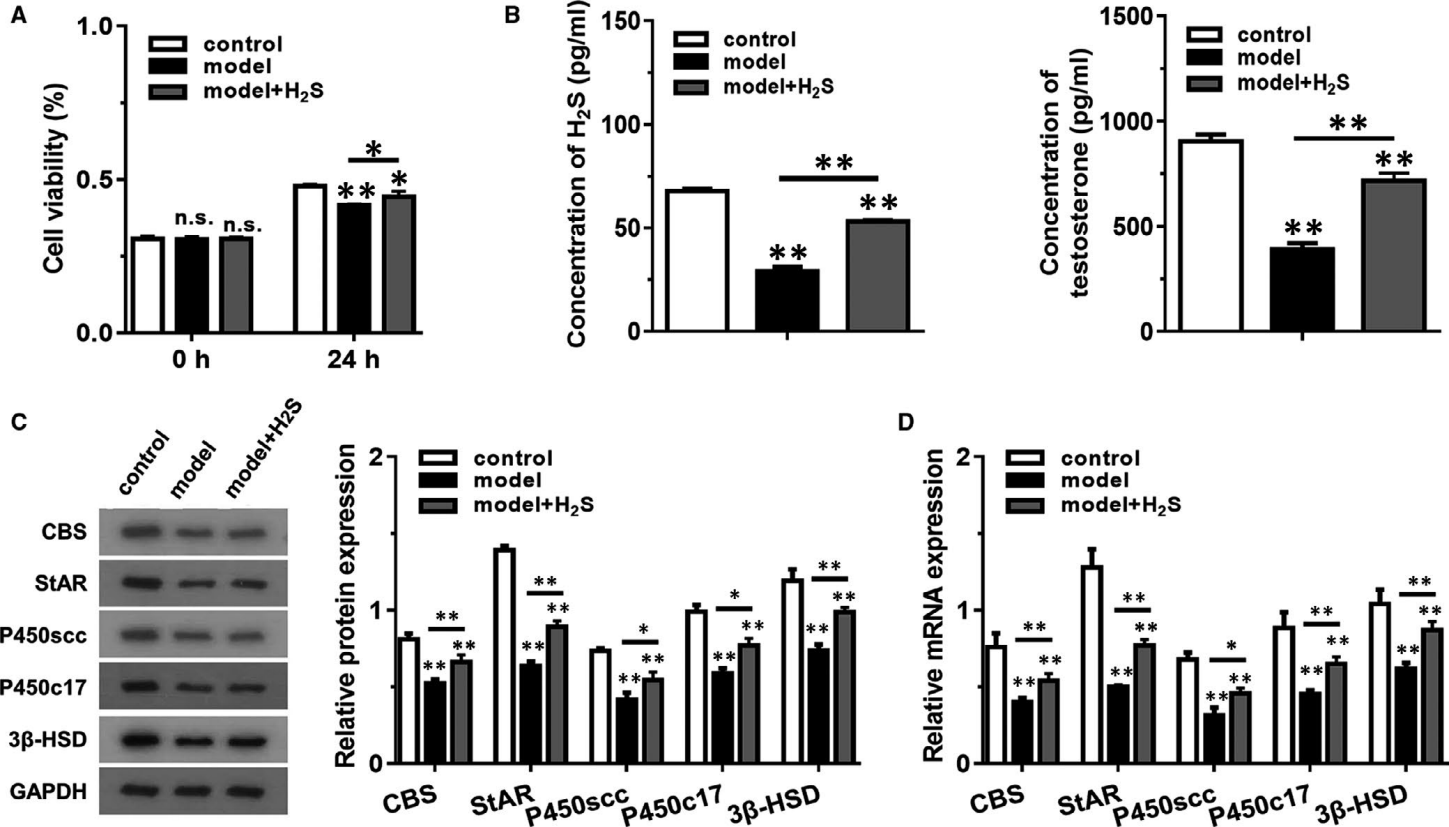
H_2_S pretreatment reduced LPS + H_2_O_2_ induced injury in testosterone synthesis of MLTC‐1 cells. The model + H_2_S group was pretreated with H_2_S donor GYY4137 (50 μmol/L) for 4 h, and then added with LPS (10 μg/mL) + H_2_O_2_ (250 μmol/L) for 12 h. The model group was pretreated with PBS and treated with LPS (10 μg/mL) + H_2_O_2_ (250 μmol/L) for 12 h, and the control group was incubated with PBS only. (A) The cell viability of each group was detected using CCK‐8 method at 0 h and 24 h. (B) The concentration of H_2_S and testosterone were examined using ELISA kits. (C) The levels of proteins related to testosterone synthesis were detected by western blotting. (D) The mRNA levels of genes related to testosterone synthesis were detected by RT‐qPCR. *P < .05, *P < .01 compared with control group, NS represented no significance

Cell viability result presented in Figure 1A revealed that LPS + H_2_O_2_ could significantly reduce viability of MLTC‐1 cells at 24 hours while pretreatment of H_2_S for 4 hours had reverse effect. As presented in Figure 1B, the concentration of H_2_S, as well as that of testosterone, were dramatically downregulated in model group compared with control group. However, the level of H_2_S and testosterone were upregulated in model + H_2_S group. Western blotting was further implemented for determining the expression of CBS, StAR, P450scc, P450c17 and 3β‐HSD (Figure 1C). The reduction of the levels of these proteins induced by LPS + H_2_O_2_ were attenuated in model + H_2_S group, suggesting that H_2_S might play a protective role in LPS + H_2_O_2_ induced injury.

### 3.2 CBS overexpression inhibited while knockdown promoted LPS + H_2_O_2_ induced injury in testosterone synthesis of MLTC‐1 cells

**FIGURE 2:**
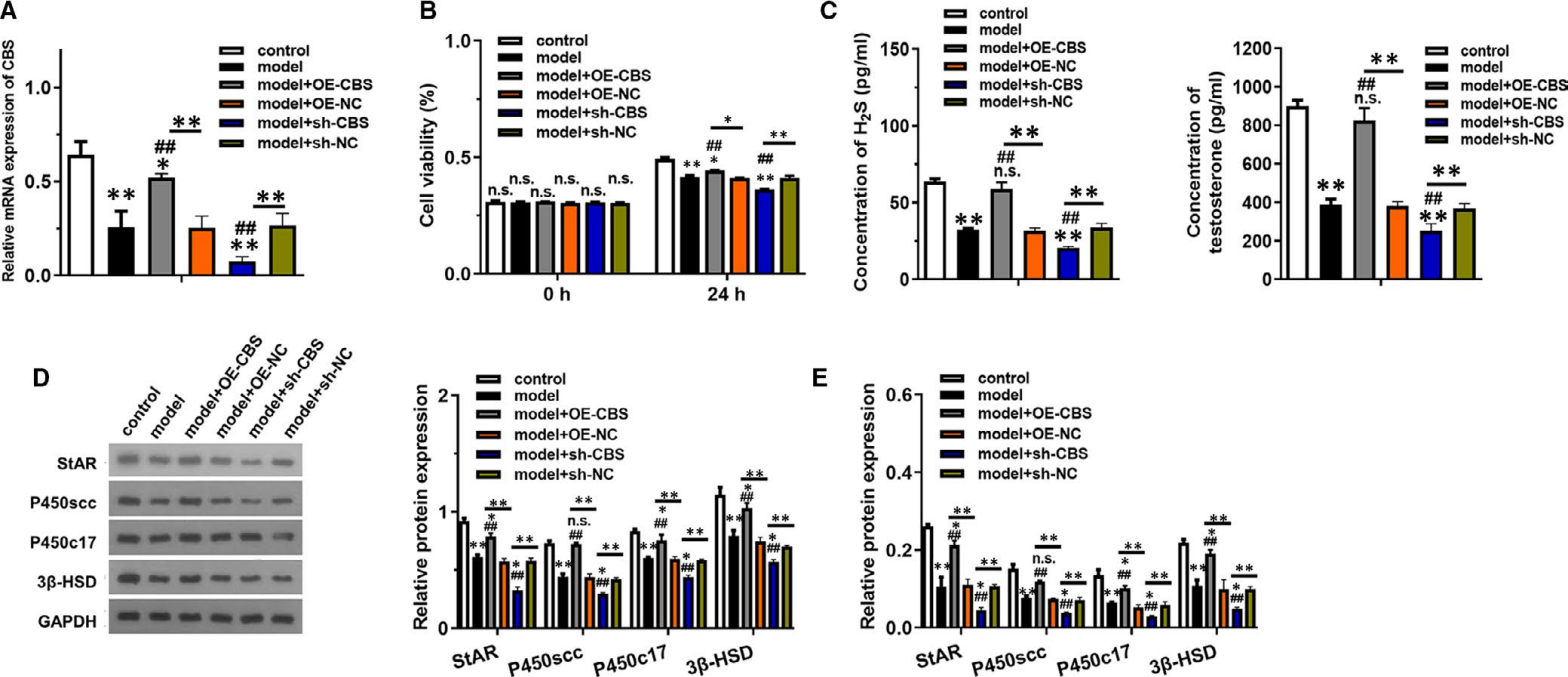
CBS overexpression inhibited while knockdown promoted LPS + H_2_O_2_ induced injury in testosterone synthesis of MLTC‐1 cells. The model group was added with LPS (10 μg/mL) + H_2_O_2_ (250 μmol/L) for 12 h, and the control group was incubated with PBS only. (A) The expression of CBS mRNA in each group treated with indicated vector transfection was detected by RT‐qPCR. (B) The cell viability of each group was detected using CCK‐8 method at 0 h and 24 h. (C) The concentration of H_2_S and testosterone were examined using ELISA kits. (D) The levels of proteins related to testosterone synthesis were detected by western blotting. (E) The mRNA levels of genes related to testosterone synthesis were detected by RT‐qPCR. *P < .05, *P < .01 compared with control group, #*P* < .05, #*P* < .01 compared with model group, NS represented no significance

To further investigate the role of CBS in LPS + H_2_O_2_ induced injury, both overexpression model and inhibited model of CBS was constructed (Figure 2A). Consistently, the cell viability was decreased in model + sh‐CBS group compared with model group, while overexpression of CBS could increase cell viability (Figure 2B). As presented in Figure 2C, the concentration of H_2_S and testosterone were both dramatically upregulated in model + OE‐CBS group compared with model group and model + sh‐CBS group. Western blotting and RT‐qPCR was employed to measure the level of StAR, P450scc, P450c17 and 3β‐HSD (Figure 2D,E). Analysis data indicated that the protein and mRNA level of StAR, P450scc, P450c17 and 3β‐HSD were all dramatically decreased in model group and model + sh‐CBS group, which was reversed in model + OE‐CBS group.

### 3.3 CBS overexpression inhibited LPS + H_2_O_2_ induced injury in testosterone synthesis through promoting sulfhydrylation of PDE4A and PDE8A

**FIGURE 3:**
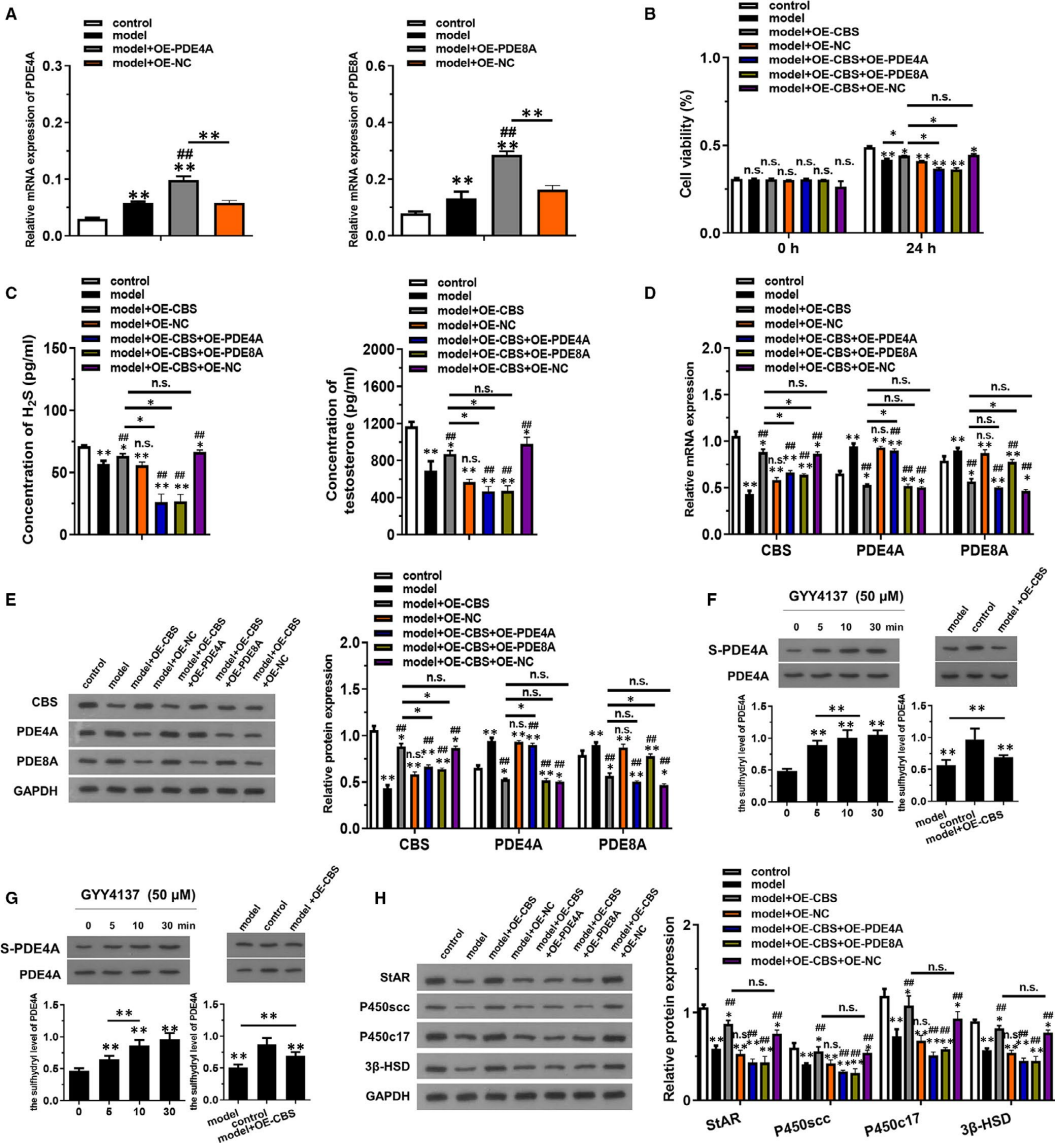
CBS overexpression inhibited LPS + H_2_O_2_ induced injury in testosterone synthesis through PDE4A and PDE8A. The model group was added with LPS (10 μg/mL) + H_2_O_2_ (250 μmol/L) for 12 h, and the control group was incubated with PBS only. (A) The mRNA expression of PDE4A and PDE8A in groups transfected with indicated vectors was detected by RT‐qPCR. (B) The cell viability of each group was detected using CCK‐8 method at 0 h and 24 h. (C) The concentration of H_2_S and testosterone were examined using ELISA kits. (D) The mRNA levels of CBS, PDE4A and PDE8A were detected by RT‐qPCR. (E) The levels of CBS, PDE4A and PDE8A proteins expression were detected by western blotting. (F and G) The sulfhydryl level of PDE4A and PDE8A was determined by Biotin Switch Technique. (H) The levels of proteins related to testosterone synthesis were detected by western blotting. *P < .05, *P < .01 compared with control group, #*P* < .05, #*P* < .01 compared with model group, NS represented no significance

PDE4A and PDE8A overexpression models were constructed to verify their relationship between CBS (Figure 3A). As shown in Figure 3B, although overexpression of CBS could attenuate LPS + H_2_O_2_ induced reduction in cell viability, simultaneously overexpression of PDE4A or PDE8A could reverse the effect of CBS. Similarly, the concentration of H_2_S and testosterone was significantly decreased in model + OE‐CBS + OE‐PDE4A and model + OE‐CBS + OE‐PDE8A group compared with model + OE‐CBS group (Figure 3C). The western blotting and RT‐qPCR results further elucidated that the originally increased protein and mRNA level of CBS was decreased after overexpression of PDE4A and PDE8A (Figure 3D,E). Biotin Switch Technique data revealed that the sulfhydryl level of PDE4A and PDE8A was increased with the H_2_O_2_ reaction time increased. The sulfhydryl level of PDE4A and PDE8A was increased in model + OE‐CBS group compared to the model group (*P* < .01). The protein level of StAR, P450scc, P450c17 and 3β‐HSD was downregulated in model + OE‐CBS + OE‐PDE4A and model + OE‐CBS + OE‐PDE8A group compared with model + OE‐CBS group (*P* < .01). These evidence demonstrated that PDE4A and PDE8A might be regulated by CBS.

### 3.4 CBS overexpression in vivo inhibited LPS + H_2_O_2_ induced injury in testosterone synthesis through activating cAMP/PKA pathway

**FIGURE 4:**
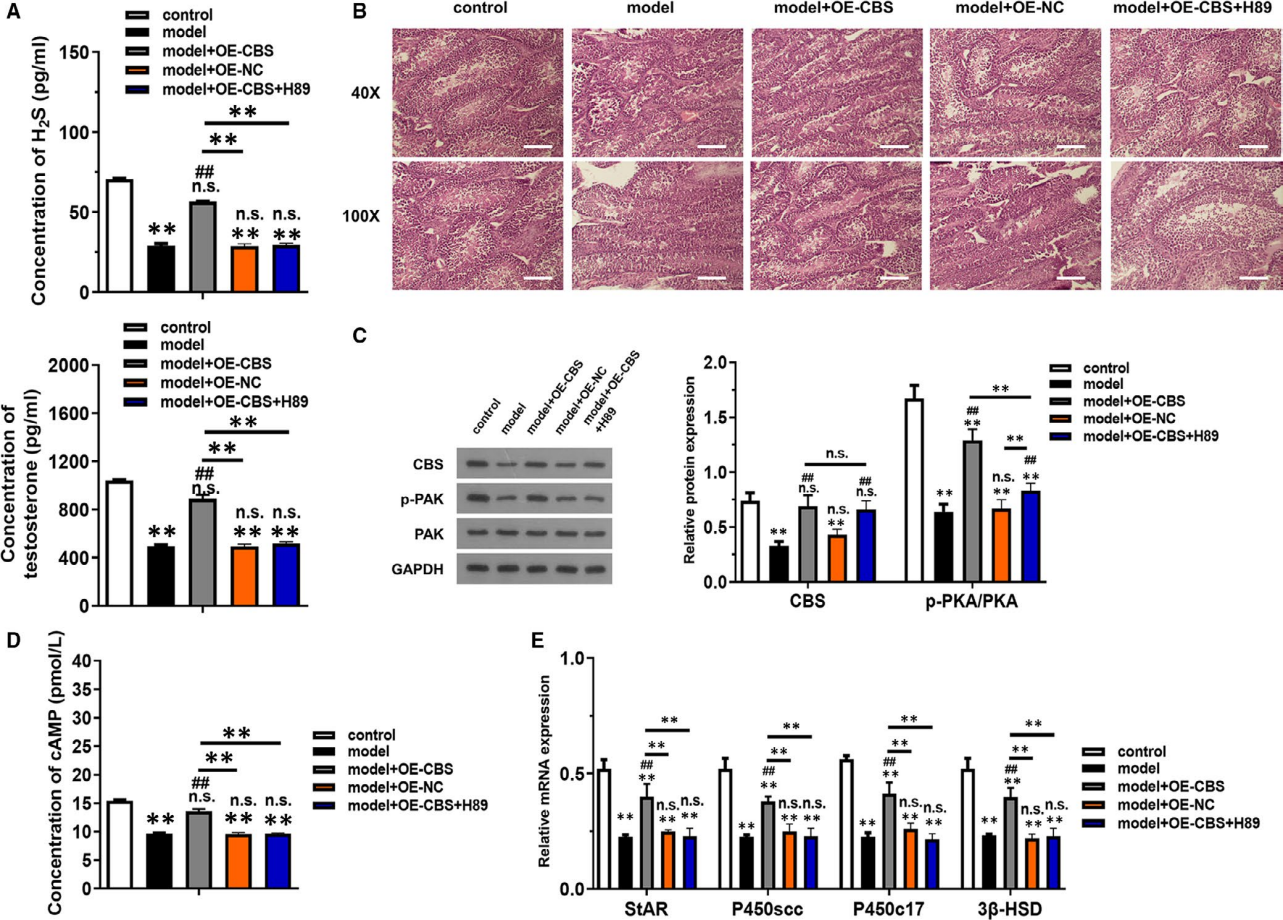
CBS overexpression in vivo inhibited LPS + H_2_O_2_ induced injury in testosterone synthesis through activating cAMP/PKA pathway. (A) The concentration of H_2_S and testosterone were examined using ELISA kits. (B) PAS staining was used to detect the inflammatory status of testis in each group. (C) The levels of CBS, p‐PKA and PKA proteins expression were detected by western blotting. (D) The activity of cAMP was examined using ELISA kits. (E) The levels of relative mRNAs related to testosterone synthesis were detected by RT‐qPCR. *P < .05, *P < .01 compared with control group, #*P* < .05, #*P* < .01 compared with model group, NS represented no significance

To further investigate the role of CBS and its potential downstream pathway cAMP/PKA in LPS + H_2_O_2_ induced injury, in vivo overexpression model of CBS was constructed. The concentration of H_2_S and testosterone was significantly increased in model + OE‐CBS compared to the model group, which was reversed by the addition of H89, the specific inhibitor of PKA (Figure 4A, *P* < .01). PAS staining showed that CBS overexpression alleviated the inflammation status and incidence of vacuoles in testis, which was totally reversed by H89 (Figure 4B). Importantly, CBS overexpression in LPS + H_2_O_2_‐induced model exerted promoted effect on the expression of p‐PKA but no influence on PKA, which indicated that CBS could activate PKA by the phosphorylation of PKA (Figure 4C, *P* < .01). The level of cAMP was decreased in model group compared to the control group, which was recovered by CBS overexpression (Figure 4D, *P* < .01). The increased effect of CBS overexpression was inhibited by H89 (Figure 4D). The mRNA level of StAR, P450scc, P450c17 and 3β‐HSD were all dramatically increased in model + OE‐CBS group, which was reversed in model + OE‐CBS + H89 group (Figure 4E).

### 3.5 CBS overexpression in vivo inhibited LPS + H_2_O_2_‐induced injury in testosterone synthesis through promoting sulfhydrylation of PDE4A and PDE8A

**FIGURE 5:**
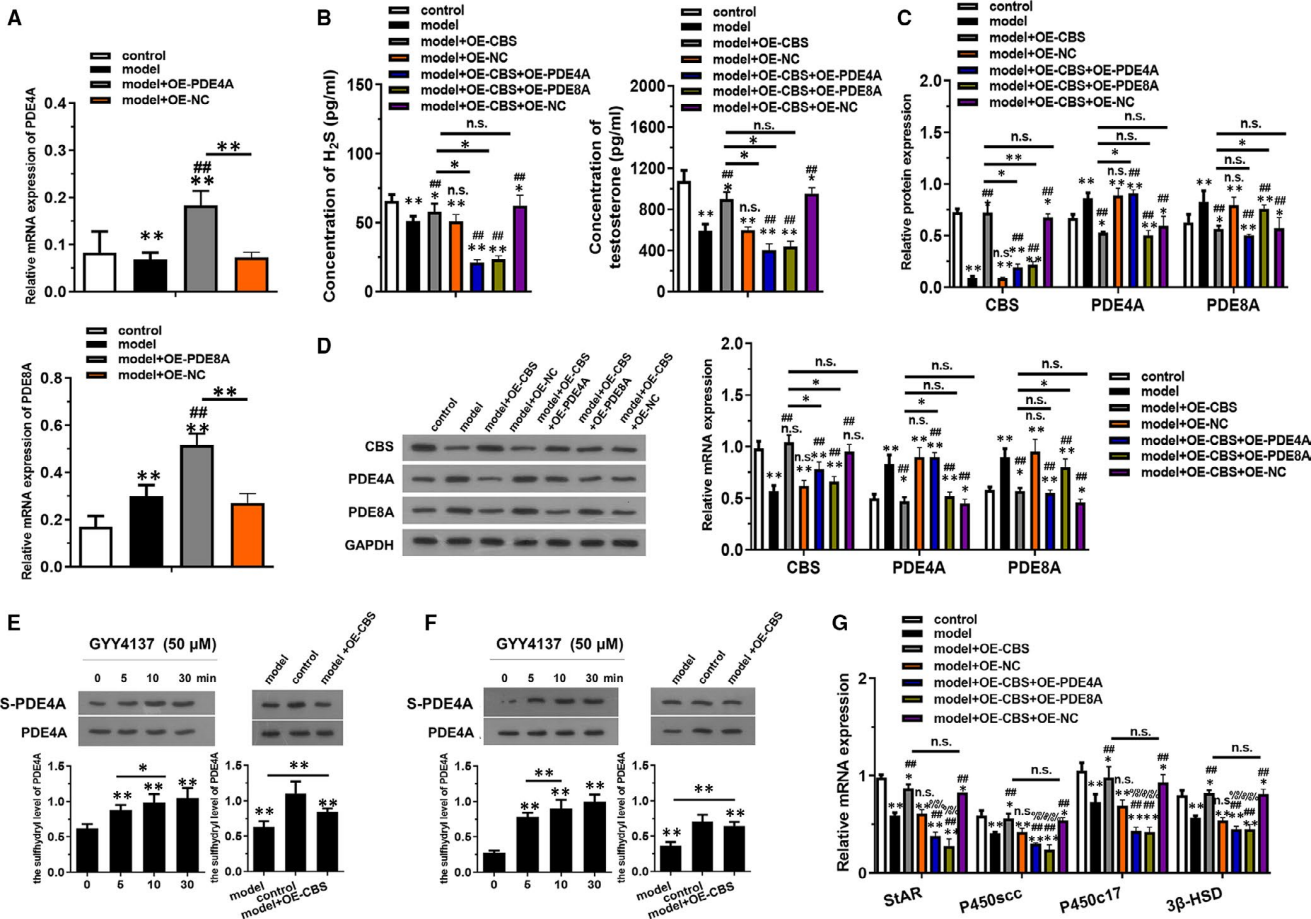
CBS overexpression in vivo inhibited LPS + H_2_O_2_ induced injury in testosterone synthesis through PDE4A and PDE8A. (A) The mRNA expression of PDE4A and PDE8A in mice transfected with indicated vectors was detected by RT‐qPCR. (B) The concentration of H_2_S and testosterone were examined using ELISA kits. (C) The mRNA levels of CBS, PDE4A and PDE8A were detected by RT‐qPCR. (D) The levels of CBS, PDE4A and PDE8A proteins expression were detected by western blotting. (E and F) The sulfhydryl level of PDE4A and PDE8A was determined by Biotin Switch Technique. (G) The levels of relative mRNAs related to testosterone synthesis were detected by RT‐qPCR. *P < .05, *P < .01 compared with control group, #*P* < .05, #*P* < .01 compared with model group, %*P* < .05, %%*P* < .01 compared with model + OE‐CBS group, NS represented no significance

PDE4A and PDE8A overexpression in vivo models were constructed to further verify their relationship between CBS (Figure 5A). As shown in Figure 5B, the concentration of H_2_S and testosterone was significantly decreased in model + OE‐CBS + OE‐PDE4A and model + OE‐CBS + OE‐PDE8A group compared with model + OE‐CBS group (*P* < .01). The RT‐qPCR and western blotting results further elucidated that the originally increased relative mRNA and protein level of CBS was decreased after overexpression of PDE4A and PDE8A (Figure 5C,D). Biotin Switch Technique data revealed that the sulfhydryl level of PDE4A and PDE8A was increased in model + OE‐CBS group compared to the model group (*P* < .01). The relative mRNA level of StAR, P450scc, P450c17 and 3β‐HSD was downregulated in model + OE‐CBS + OE‐PDE4A and model + OE‐CBS + OE‐PDE8A group compared with model + OE‐CBS group (*P* < .01). These evidence demonstrated that CBS overexpression could inhibit PDE4A and PDE8A through promoting sulfhydrylation of PDE4A and PDE8A.

## 4 DISCUSSION

Testosterone deficiency exhibits negative impact on male reproductive and sexual functions, and one of the common causes of testosterone deficiency was testosterone synthesis affected by gene, ageing or infections of male reproductive system, which are universally accompanied by inflammatory and oxidative damage.^14^, ^15^, ^16^ Improving the synthesis of endogenous testosterone may be of important implications for reducing the potential risks associated with androgen replacement therapy.^17^, ^18^

The classical molecular signalling pathway for testosterone synthesis is luteinizing hormone/luteinizing hormone receptor/cyclic adenosine monophosphate/protein kinase A (LH/LHR/cAMP/PKA). Activation of the PKA signalling pathway causes the initiation of a variety of transcription factors that regulate testosterone synthesis by regulating the expression of related proteins, including StAR, P450scc, P450c17 and 3β‐HSD.^19^, ^20^ CAMP is a fairly important second messenger in the testosterone synthesis LH pathway, and PDE is the key enzyme for the degradation of cAMP.^21^, ^22^ Several studies have shown that H_2_S is a non‐specific inhibitor of PDE and participates in the regulation of cAMP‐dependent signalling pathways.^7^ PDE, as a drug target, has received extensive attention for a long time.^23^ Numerous studies have confirmed that PDE could regulate testosterone synthesis in MLTC‐1 cells, and inhibitors of PDE could increase the level of testosterone synthesis in MLTC‐1.^24^, ^25^, ^26^ In the cardiovascular system, H_2_S can increase the levels of cAMP by inhibiting the activity of PDE in the aortic ring, and then regulate cAMP‐dependent vasodilation.^27^ H_2_S can activate electron transport and energy metabolism in rat liver mitochondria, and its main mechanism is that H_2_S can inhibit the activity of PDE2A and activate cAMP/PKA‐dependent cellular energy metabolism.^28^ However, the significance of H_2_S in male reproduction has not been clear.

Our previous work found that H_2_S could significantly reduce the spermatogenesis disorder caused by inflammation and oxidative stress, among which H_2_S from CBS might be one of the key factors to maintain testicular function.^9^ In this study, we further investigated the effect of H_2_S caused by CBS overexpression or inhibition on PDE4A and PDE8A in vitro and in vivo, and their relationship was elucidated. PDE4A and PDE8A are the most important PDE subtype regulating cAMP‐dependent testosterone synthesis in MLTC‐1 cells ^29^ since they participate in the regulation of cAMP levels in mitochondria and cytoplasm. We found that CBS overexpression could recover the LPS + H_2_O_2_ induced testosterone biosynthesis damage by combined inhibition of high expression of PDE4A and PDE8A both in vivo and in vitro. Also, the level of cAMP and p‐PKA was increased by CBS overexpression, which could be reversed by H89, the specific inhibitor of PKA. Therefore, H_2_S catalysed by CBS could inhibit the expression of PDE4A and PDE8A, which was of great significance to solve the disorder of testosterone synthesis.

Finally, we investigated the mechanism by which H_2_S downregulated the expression of PDE4A and PDE8A. Using BST method, the inhibitory effects that H_2_S exhibited resulted from its modification of PDE, since sulfhydryl level of PDE4A and PDE8A was increased with CBS overexpression or H_2_S reaction time promoted. Other recent studies have also confirmed that H_2_S could modify PDE through sulfhydryl modification, and then increase cAMP level.^30^ Hence, H_2_S played a physiological role on downregulating PDE level via sulfhydrylation. However, the present study only investigated the sulfhydryl modification of H_2_S on PDE. The other molecules regulated by H_2_S are needed to be explored in the future experiments to elucidate the comprehensive mechanisms of H_2_S in the disorder of testosterone synthesis.

## 5 CONCLUSION

H_2_S catalysed by CBS could recover testosterone synthesis in vitro and in vivo through inhibiting PDE expression via sulfhydryl modification of PDE, and activating cAMP/PKA pathway, which provided a theoretical basis for endogenous regulation in the treatment of testosterone deficiency.

## CONFLICT OF INTEREST

The authors declare that they have no competing interests.

## AUTHOR CONTRIBUTIONS

**Jing Wang:** Conceptualization (equal); data curation (equal); formal analysis (equal); funding acquisition (equal); methodology (equal); project administration (equal); resources (equal); software (equal); supervision (equal); validation (equal); visualization (equal); writing‐original draft (equal); writing‐review & editing (equal). **Jing Wang**: Data curation (equal); formal analysis (equal); investigation (equal); methodology (equal); resources (equal); software (equal); supervision (equal); validation (equal); visualization (equal). **Tao Shen**: Data curation (equal); formal analysis (equal); investigation (equal); methodology (equal); software (equal); supervision (equal); validation (equal). **Renyun Hong**: Data curation (equal); formal analysis (equal); investigation (equal); software (equal); supervision (equal); validation (equal); visualization (equal). **Shanshan Tang**: Formal analysis (equal); investigation (equal); software (equal); supervision (equal); visualization (equal). **Xia Zhao**: Formal analysis (equal); investigation (equal); methodology (equal); software (equal); supervision (equal).

## ETHICAL APPROVAL

The research protocol was reviewed and approved by the Ethical Committee and Institutional Review Board of the Zhongda Hospital, School of Medicine, Southeast University.

## CONSENT TO PUBLISH

All of the authors have consented to publish this research.

## Data Availability

The data are free access to available upon request.
